# Suave-Kapandji Reconstruction After Excision of the Distal Ulna for Chondrosarcoma: A Case Report

**DOI:** 10.7759/cureus.31358

**Published:** 2022-11-11

**Authors:** Clarice Callahan, Jordan R Nester, Thomas R Bowen

**Affiliations:** 1 Orthopedic Surgery, Geisinger Medical Center, Danville, USA; 2 Education, Michigan State University College of Human Medicine, Grand Rapids, USA; 3 Orthopedic Oncology, Geisinger Medical Center, Danville, USA

**Keywords:** pathological fracture, druj, distal ulna, chondrosarcoma, suave-kapandji

## Abstract

An active, 62-year-old man presented with a nondisplaced pathological fracture through a low-grade, central chondrosarcoma of the distal ulnar diaphysis after minor trauma. After obtaining diagnostic imaging, the patient was successfully treated with marginal en-bloc resection of the right distal ulnar diaphysis and wrist reconstruction via a Sauve-Kapandji arthroplasty. Suave-Kapandji arthroplasty is an alternative reconstruction to complete the excision of the distal ulna following resection of the distal ulnar diaphysis.

## Introduction

Surgery is the mainstay of treatment for most symptomatic cartilaginous tumors, with very limited roles for chemotherapy and radiation in selected cases [1-3]. Wide excision is used for most malignant cartilaginous neoplasms and curettage is reserved for symptomatic benign lesions [1,4]. Recognizing a spectrum of the malignant phenotype of cartilaginous neoplasms and its implications for treatment, the fifth edition of the WHO Classification of Tumours proposes the term “central atypical cartilaginous tumor” instead of “chondrosarcoma, grade 1” for low grade, medullary cartilaginous neoplasms of the appendicular skeleton [5]. It is recognized that these lesions have some risk of local recurrence following surgical treatment, but lack metastatic potential in the absence of progression to a higher grade [1,3,6]. This can produce a surgical dilemma due to the need to control local symptoms and decrease the risk of progressive disease and local recurrence, while also recognizing the limited metastatic potential and excellent overall survival [1,4,5].

In the case report presented, we describe low-grade chondrosarcoma of the distal ulna. Chondrosarcoma is one of the most frequently occurring bone sarcomas with an incidence of ~0.1/100,000 per year [5]. Chondrosarcomas mostly arise in long bones, but can also develop in flat bones. Ulnar involvement of primary bone tumors and tumor-like lesions, however, is extremely rare and is reported to only consist of 1% of all lesions [7]. Due to the paucity of these neoplasms in the distal ulna, there are multiple acceptable treatment options without one proven to be superior to the others. Treatment of this low-grade chondrosarcoma of the distal ulna with extended intralesional curettage would likely result in good local control and a modest risk of local recurrence. However, the lesion occurs in an "expendable bone" and can be treated with marginal en-bloc excision. Compared to curettage, marginal en-bloc excision would provide a much-reduced risk of local recurrence, while maintaining a good functional outcome [8]. Previous critics of distal ulna excision note in prior studies that potential outcomes however could include weakness of grip, loss of ulnar support at the carpus, ulnar translation of the carpus, radioulnar impingement, and pronounced radioulnar instability [9].

Marginal en-bloc resection of the right distal ulnar diaphysis does not routinely create the need for reconstruction due to the preservation of function. If reconstruction is desired for cosmetic purposes or to maintain the integrity of the ulnar wrist joint, there are no standard reconstruction options currently described. Historically, the Suave-Kapandji procedure is used as a salvage operation for the management of an altered distal radioulnar joint (DRUJ) [10]. This technique involved arthrodesis of the DRUJ and segmental resection of the distal ulna; this provides preservation of the ulnar support of the wrist while still allowing for shortening of the ulnar head [10]. Suave-Kapandji could be considered for reconstruction in this case.

## Case presentation

**Figure 1:**
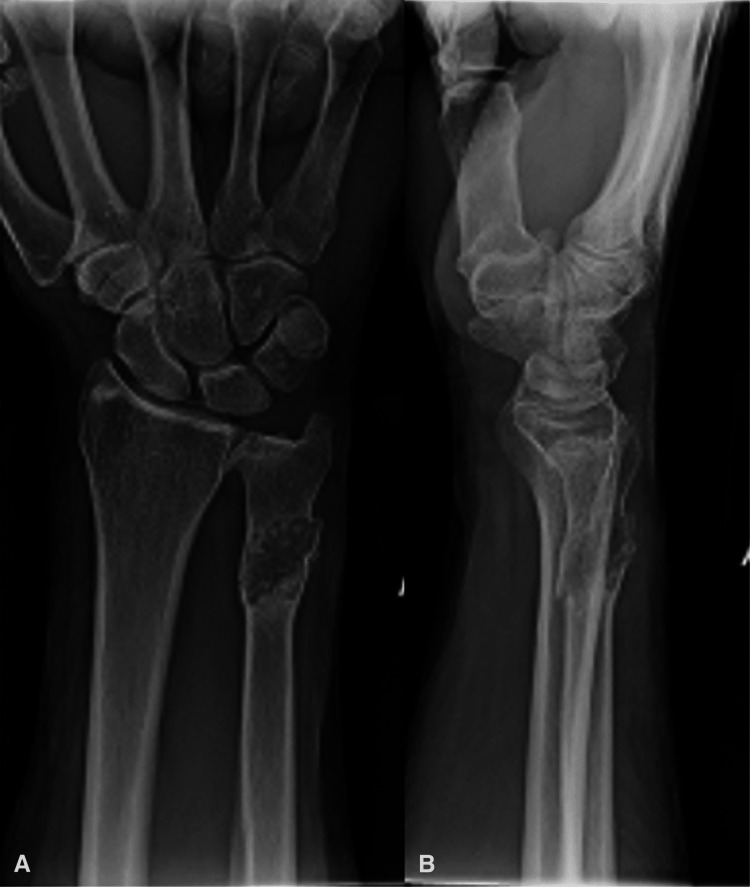
Pre-operative X-ray of the right forearm. Frontal (A) and lateral (B) view. Shows a comminuted, pathologic fracture of the right distal ulna through a lucent lesion with matrix mineralization.

The patient was a 62-year-old man with no history of neoplasia, except for basal cell carcinoma of the skin, who presented with complaints of right wrist pain after striking his hand on a door while moving boxes at work. He reported no prodromal wrist symptoms and no history of injury or surgery involving the right upper extremity. He was a current smoker, of about ½ packs per day for the past 30 years, and had been diagnosed with chronic obstructive pulmonary disease (COPD). On physical exam, the patient had mild ecchymosis about the distal, ulnar forearm, without open injury. There was tenderness to palpation over the distal aspect of the right ulna with attempted active pronation and supination of the forearm, but no tenderness in the hand or proximally in the right upper extremity. The sensation and motor function in the hand were intact. Radiographs of the forearm demonstrated a pathologic fracture of the distal ulna through a 1 cm x 2 cm centrally located, lytic lesion of the distal 1/3 of the diaphysis (Figure 1). The lesion contained a mineralized matrix suggestive of a cartilaginous neoplasm. There was deep endosteal scalloping extending to the periosteum in some locations. There was no periosteal reaction. 

**Figure 2:**
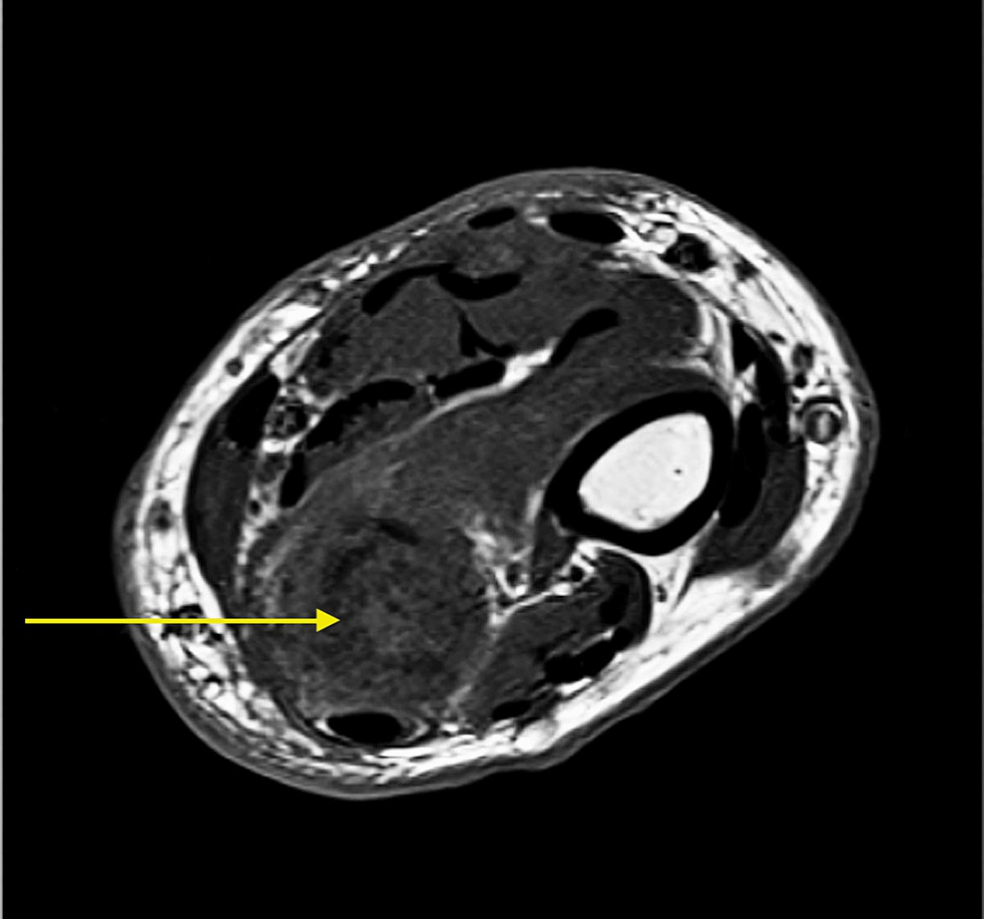
Pre-operative T1 weighted MRI of the right forearm in axial view. Shows complete displacement of marrow fat with tumor and severe cortical thinning.

**Figure 3:**
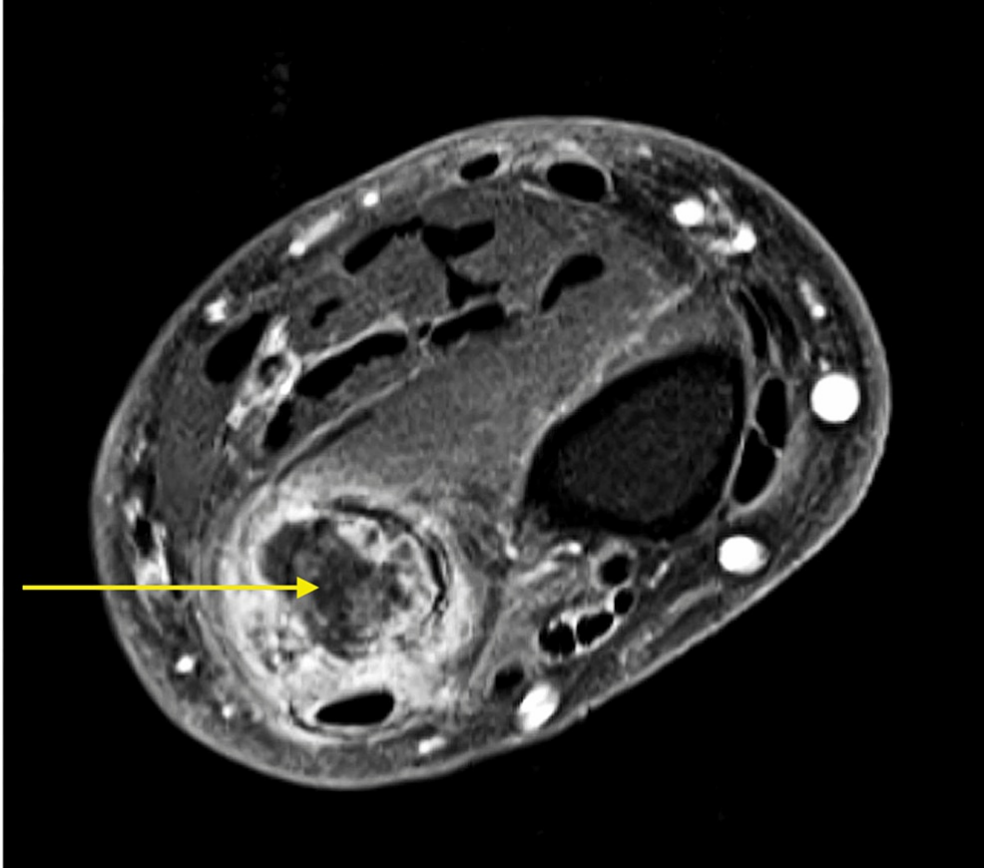
Pre-operative T1 weighted MRI of the right forearm with fat saturation post-contrast in axial view. Shows lobular enhancement, which is diagnostic for a cartilage tumor.

An MRI of the lesion was obtained and again demonstrated the distal ulnar bone tumor with cartilaginous matrix and pathological fracture (Figure 2, 3). There was no soft tissue mass. Screening plain radiographs and chest CT were negative for lung neoplasm. Surgical intervention was recommended with a plan for marginal en bloc excision of the distal ulnar diaphysis and Suave-Kapandji reconstruction.

Surgical technique

A longitudinal incision was made along the subcutaneous border of the right distal ulna. The distal ulna was circumferentially exposed with the maintenance of the periosteum and adjacent soft tissues in continuity with the tumor. By direct measurement, and with fluoroscopic confirmation, we performed transverse osteotomies of the distal ulna, 1 cm proximal from the tip of the ulnar styloid and proximal to the bone lesion using a micro sagittal saw. The intermuscular septum was then transected leaving approx. 2 mm tissue attached to the lesion to serve as a margin. A “marrow margin” was obtained proximally with a small curette but was not able to be obtained distally as the remaining distal ulnar segment had no medullary canal. Intraoperative inspection of the lesion in the surgical pathology laboratory demonstrated close, but grossly negative margins. The frozen section of the proximal “marrow margin” and touch prep of the distal osteotomy site of the specimen were negative for tumor.

The Suave-Kapandji reconstruction was performed by rotating the remaining distal ulna away from the radius to expose the distal radial ulnar joint (DRUJ) through the tumor bed and the proximal DRUJ joint. The ulnar wrist soft tissue attachments to the distal ulna and articular surface were not exposed. The DRUJ articular surface was completely removed with a curette and a small rongeur. The DRUJ was reduced under direct vision and stabilized with two 4.0 mm cannulated screws.

**Figure 4:**
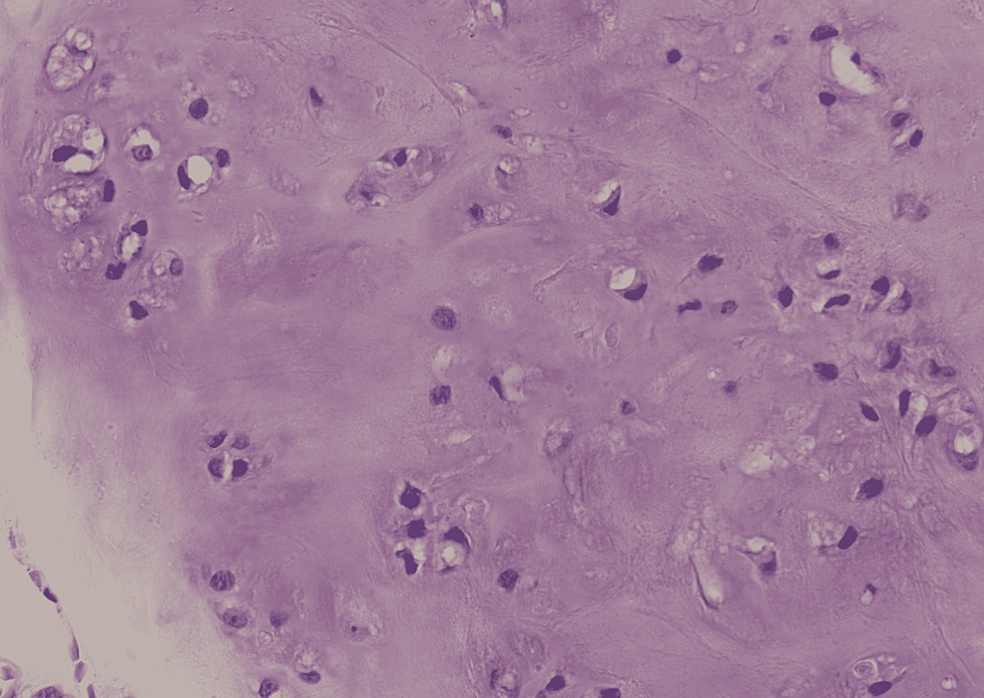
Histological features of the low-grade chondrosarcoma at magnification 400x. Chondrosarcoma shows mild cellularity, with cells embedded in a hyaline cartilaginous matrix. Nuclei show condensed chromatin.

**Figure 5:**
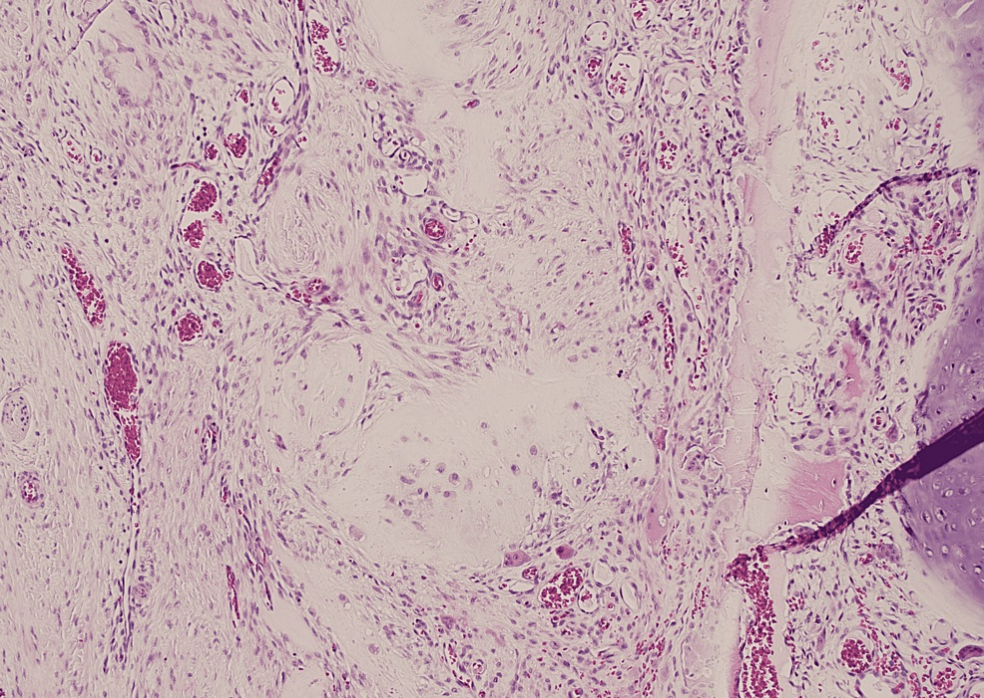
Histological features of the low-grade chondrosarcoma at magnification 200x. Chondrosarcoma demonstrating permeation through periosteum into surrounding soft tissue.

Final surgical pathology demonstrated a 2.5 x 1.5 x 1.2 cm white-grey lesion within the distal ulna. Histological analysis showed a hyaline cartilage neoplasm with a lobular architecture, no significant increase in cellularity, and no cytological atypia. The tumor penetrated the cortex multifocally, resulting in a prominent periosteal reaction. The histological features, reviewed in context with the preoperative imaging, were consistent with low-grade chondrosarcoma (Figure 4, 5). All surgical margins were negative (no tumor on ink). The distal bone margin was 0.2 cm from the lesion, as were multiple surrounding soft tissue margins.

**Figure 6:**
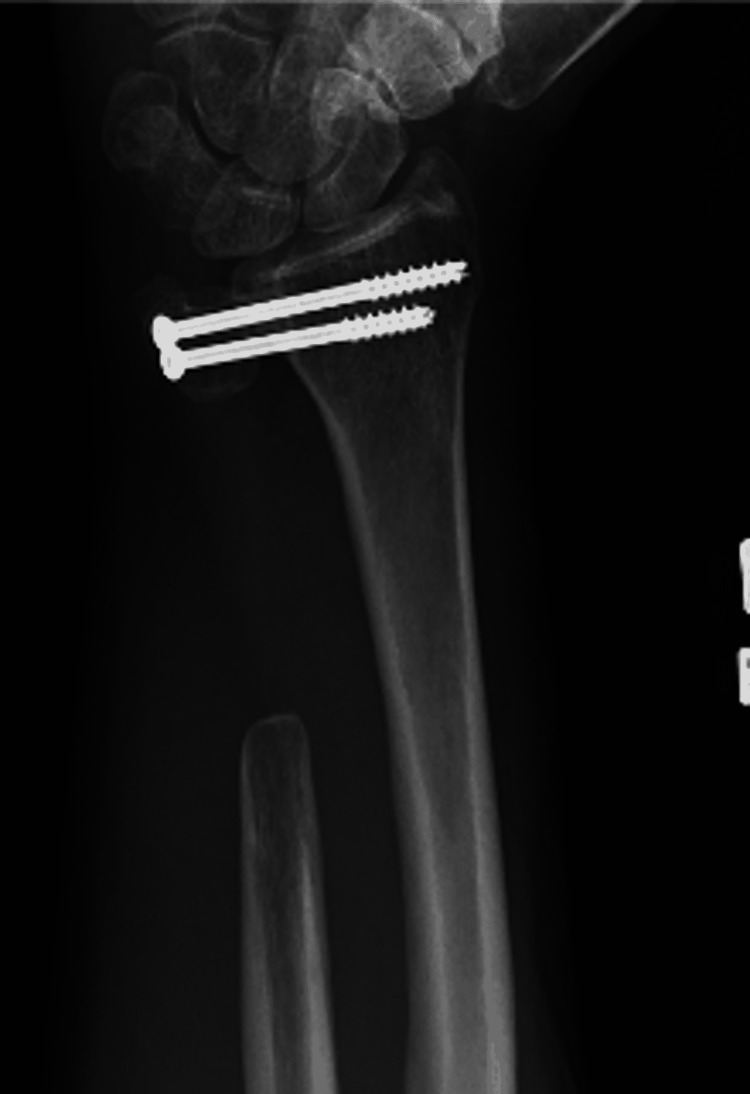
X-ray of the right forearm taken two years after the initial pre-operative X-ray. Shows prior resection of the right distal ulna with two intact metallic screws spanning the distal radioulnar joint. Overall stable postoperative changes with no findings to suggest recurrent disease.

**Figure 7:**
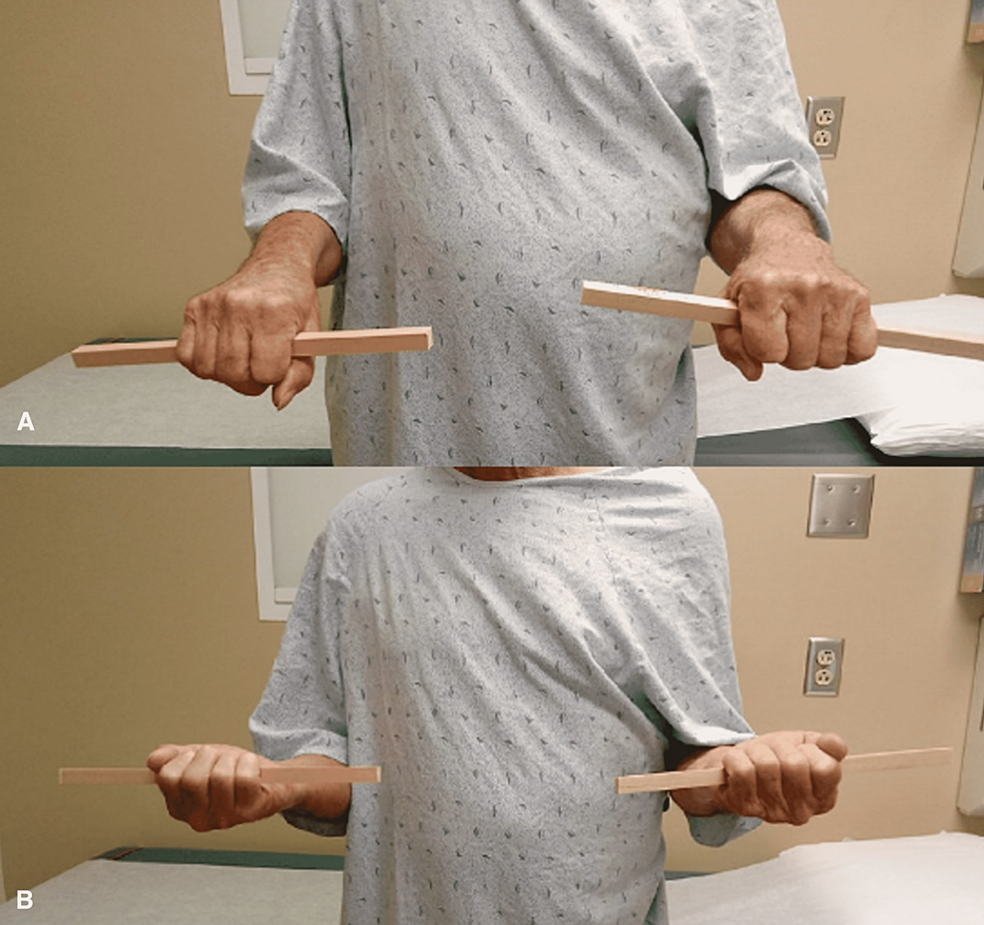
Patient post-operatively. Demonstrating fully intact and symmetric pronation (A) and supination (B) bilaterally.

Three months after surgery, the patient had minimal complaints. His right wrist motion was symmetric to the contralateral side in flexion/extension as well as pronation and supination. At 12 months after surgery, the patient had no complaints of pain and had maintained a symmetric range of motion to his contralateral side; this continued through his 24-month post-operative appointment as well (Figure 6, 7). He continues to have no limitations when using his right upper extremity and has no evidence of local recurrence or metastatic disease to date.

## Discussion

The distal ulna is an “expendable bone,” routinely resected for malignant bone tumors without reconstruction and with good functional outcomes [11]. In the presented case, the diaphyseal location and low-grade nature of the tumor necessitated only a marginal en bloc excision for local control of the tumor. This allows theoretical preservation of the distal ulna and the ulnar wrist articular soft tissues. The senior author (Thomas R. Bowen) initially recommended excision of the entire distal ulnar without reconstruction, per routine; however, the patient requested the preservation of the distal ulnar fragment, to maintain the cosmetic contour of the wrist and maintain the integrity of the ulnar wrist joint and ligamentous structures. The patient’s request prompted the consideration of alternative reconstruction options. To the authors’ knowledge, this is the first reported use of the Suave-Kapandji reconstruction for orthopedic oncological reconstruction.

Historically, wide excision without chemotherapy or radiation has been the most common treatment for central, low-grade chondrosarcoma of the long bones of the extremities [5]. Excellent local tumoral control was achieved, but with considerable musculoskeletal morbidity [1]. In recent decades, an increasing appreciation for the near-absent metastatic potential for central, low-grade chondrosarcoma of the extremities has led to most of these lesions being treated with extended intralesional excision with high-speed burr and other adjuvants, rather than wide excision [12]. Extended intralesional excision tolerates a modest risk of local recurrence in exchange for reduced functional disability and reduced surgical complication risk. Excision, usually in a marginal en bloc fashion, remains a common treatment for “expendable bones,” such as the ribs, clavicle, distal ulna, iliac crest, scapular body, and proximal fibula. In these locations, the morbidity of wide excision is small and musculoskeletal function remains excellent without reconstruction [8].

## Conclusions

In the present case, complete excision of the tumor, including the remaining distal ulna would likely have provided good functional outcomes with a low complication risk. However, the patient was disappointed in the potential cosmetic loss of the distal ulna and wished to avoid what he perceived as unnecessary manipulation of the wrist joint. The authors propose the Suave-Kapandji reconstruction as an alternative to complete excision of the distal ulna in select patients, with low additional risk of complication and avoidance of unnecessary manipulation of the ulnar wrist.
